# Prognostic value of RDW alone and in combination with NT‐proBNP in patients with heart failure

**DOI:** 10.1002/clc.23850

**Published:** 2022-05-27

**Authors:** Lin Liang, Liyan Huang, Xuemei Zhao, Lang Zhao, Pengchao Tian, Boping Huang, Jiayu Feng, Jian Zhang, Yuhui Zhang

**Affiliations:** ^1^ State Key Laboratory of Cardiovascular Disease, Heart Failure Center, Fuwai Hospital, National Center for Cardiovascular Diseases Chinese Academy of Medical Sciences & Peking Union Medical College (CAMS & PUMC) Beijing China; ^2^ Key Laboratory of Clinical Research for Cardiovascular Medications National Health Committee Beijing China

**Keywords:** biomarkers, heart failure, NT‐proBNP, prognosis, red cell distribution width

## Abstract

**Background:**

Red blood cell distribution width (RDW) and N‐terminal pro brain natriuretic peptide (NT‐proBNP) may predict the prognosis of heart failure (HF). However, the impact of combined RDW and NT‐proBNP levels as a prognostic marker of HF remains unclear and the significance of this combination at various time‐points has not been sufficiently studied.

**Hypothesis:**

RDW can predict prognosis in HF at various time‐points and combination with NT‐proBNP improves the prognostic value.

**Methods:**

Patients admitted to HF care unit of Fuwai Hospital CAMS&PUMC (Beijing, China) with a diagnosis of HF from November 2008 to November 2018 were analyzed retrospectively.

**Results:**

In total, 3231 patients with available RDW data at admission were evaluated (median age 58 years, 71.9% males, 39.7% coronary heart disease, 68.6% New York Heart Association [NYHA] III or IV). Median RDW and NT‐proBNP at admission were 13.4% (interquartile range [IQR]: 12.7%–14.5%), and 1723.00 pg/ml (IQR: 754.00–4006.25 pg/ml), respectively. During 2.9‐year median follow‐up, all‐cause death occurred in 1075 (33.27%) patients. Kaplan–Meier survival curve and Cox proportional‐hazard models, showed patients in the top quarter RDW had a 32.0% increased mortality compared to the bottom quarter (hazard ratio: 4.39, 95% confidence interval: 3.59–5.38; *p* <.001). The top quarter RDW retained independent prognostic value across HF with reduced ejection fraction [HFrEF], HF with mid‐range ejection fraction [HFmrEF], and HF with preserved ejection fraction [HFpEF] subgroups. Patients were subsequently divided into four groups by median RDW and NT‐proBNP. Comparison of Kaplan–Meier survival curves for various groups showed good risk stratification (*p* < .001).

**Conclusions:**

RDW is an independent predictor of mortality among patients with HF in the short‐, medium‐, and long‐term. Combination of RDW and NT‐proBNP improves the prognostic value. This is true across all clinical subtypes of heart failure (HFrEF, HFmrEF, HFpEF), and among most subgroups of patients with various comorbidities (infection, diabetes, hypertension).

## 1 INTRODUCTION

Heart failure (HF), the last bastion of cardiovascular disease, is associated with high morbidity, mortality, and rates of hospitalization.^1^, ^2^, ^3^, ^4^ Predicting the risk of death or hospitalization in HF patients may direct decisions on the appropriateness and timing of treatment.^5^, ^6^ In addition, identifying predisposing factors for mortality or hospitalization may reveal targets for intervention.^5^, ^6^ Many prognostic biomarkers, including natriuretic peptide (B‐type natriuretic peptide [BNP] and N‐terminal pro brain natriuretic peptide [NT‐proBNP]) have been well‐studied in HF patients, but their clinical application is limited due to challenges in risk stratification.^7^ Red blood cell distribution width (RDW), an inexpensive and convenient parameter, is reportedly a powerful predictor of prognosis in HF patients.^8^, ^9^

However, the impact of combined RDW and NT‐proBNP levels as a prognostic marker of HF remains unclear. The significance of this combination at various time‐points has not been sufficiently studied. Also, prognostic implications of RDW in subsets of HF patients, such as HF with preserved ejection fraction (HFpEF), and in subgroups with certain comorbidities (diabetes, hypertension) remains unknown.

This study aimed to verify the role of RDW in predicting short‐, medium‐, and long‐term prognosis in HF patients within various left ventricular ejection fraction (LVEF) categories, and to evaluate its utility when combined with NT‐proBNP.

## 2 METHODS

### 2.1 Study sample

We prospectively enrolled 5124 patients admitted to HF care unit of Fuwai Hospital CAMS&PUMC (Beijing, China) with a clinical diagnosis of HF from November 2008 to November 2018. Data underwent exploratory retrospective analysis.

HF was diagnosed according to the Chinese HF Diagnosis and Treatment Guidelines.^10^ Diagnosis was confirmed by two cardiologists. For patients hospitalized more than once, data from the first admission were used. Patients were excluded if they had severe renal impairment (estimated glomerular filtration rate [eGFR] < 30 ml/min/1.73 m^2^), severe hepatic dysfunction (total bilirubin > 3.0 mg/dl), ongoing non‐CHF inflammatory processes (e.g., severe arthritis, inflammatory bowel disease, Bechet's disease, Sjogren's syndrome), an underlying condition associated with increased RDW (i.e., hemolytic anemia, sickle cell disease, thalassemia, hereditary spherocytosis, aplastic anemia, or myelodysplastic syndrome), active malignancy, pregnancy, gastric or duodenal ulcer, recent transfusion or use of iron or erythropoietin (within past 3 months).

Among the 4346 patients enrolled with available RDW data, 1115 were met exclusion criterion (*n* = 654) or were lost to follow‐up (*n* = 461, 10.6%). The final analytical cohort included 3231 individuals (Supporting Information: Figure S1).

Informed consent was obtained from all subjects. The study was in line with the Declaration of Helsinki and approved by the ethics committee of Fuwai Hospital. Patients or the public were not involved in the design, conduct, reporting, or dissemination plans of our research.

### 2.2 Baseline study variable measurements

At admission, blood samples were collected at 6 a.m. and processed immediately at the clinical laboratory of Fuwai Hospital. The reference range of RDW was 0.0%–15.0%. Cardiac function was measured by transthoracic echocardiogram (EPIQ 7G; Philips HealthTech). LVEF was calculated using the modified Simpson method detailed in the American Society of Echocardiography and the European Association of Cardiovascular Imaging guidelines.^11^ Comorbidities (hypertension and diabetes mellitus) were diagnosed according to the World Health Organization International Classification of Disease.

### 2.3 Clinical follow‐up

Primary endpoints were all‐cause mortality or cardiovascular mortality, and all study participants were followed from discharge. Mortality data were collected from routine follow‐up by outpatient visits or phone calls at 1, 6, and 12 months, and then yearly.

## 3 STATISTICAL ANALYSES

Continuous data were evaluated for normality using histograms. Normally distributed variables were presented as mean (standard deviation [SD]), whereas nonnormal variables were presented as median (interquartile range [IQR]). Categorical variables were expressed as frequencies (%). NT‐proBNP was log_2_‐transformed because of its right‐skewed distribution.

Patients were divided into four groups based on RDW quartiles. *χ* ^2^ test or one‐way analysis of variance tested for differences in categorical or continuous variables as appropriate. Nonparametric equivalents were used as appropriate (Fisher's exact test, Kruskal–Wallis test).

Survival analysis was performed for all‐cause and cardiovascular mortality. Time zero was defined as the date of hospital admission. Kaplan–Meier curves were generated for each RDW quartile. Survival was estimated by the Kaplan–Meier method, and differences in survival were evaluated with a stratified log‐rank test.

Cox proportional‐hazard models were constructed to explore the relationship between variables and all‐cause mortality, with follow‐up beginning on the date of hospital admission. Models were adjusted for age, sex, and clinical variables associated with adverse clinical outcomes. The prognostic value of RDW was evaluated before and after adjustment of potential confounders. A *p* < .05 was considered significant. The Schoenfeld residuals test was used to test the proportional hazard assumption in a Cox model. This modeling was also performed separately for time to 6‐month all‐cause mortality, 1‐year all‐cause mortality, and 2‐year all‐cause mortality to account for relationship between RDW and short‐ and medium‐ term outcomes.

Cubic spline interpolation was used to represent changes in risk according to RDW values. The RDW value for which hazard ratio (HR) = 1 was chosen as the value corresponding to the inflection point of the curve (i.e., the point above which the slope of the curve becomes steeper). Two‐tailed *p* < .05 was considered significant. Cubic spline interpolation was also evaluated after adjustment for potential confounders.

To illustrate the incremental power of including both NT‐proBNP and RDW, patients were divided into four groups according to the median RDW (13.4%) and NT‐proBNP (1737.00 pg/ml). The log‐rank test (Mantel–Cox) was used to compare survival times on Kaplan–Meier curves. The incremental value of RDW in addition to NT‐proBNP, and other potential confounders, in predicting all‐cause mortality was calculated using Harrell's C statistic.

Furthermore, to quantify the predictive accuracy of RDW for all‐cause mortality at any time period, receiver‐operating characteristics curves were plotted and areas under the curves (AUCs) were calculated. The optimal cutoffs for receiver‐operating characteristics curves were established by Youden's J statistics. The DeLong's test was used to compare the receiver‐operating characteristic curves for RDW and NT‐proBNP.

IBM SPSS Statistics version 25 (IBM) and R statistical software version 3.6.2 (R Foundation) were used for all statistical analyses.

## 4 RESULTS

### 4.1 Baseline characteristics

Supporting Information: Table S1 describes the baseline clinical characteristics of the final analytical cohort (*n* = 3231) relative to the original population (*n* = 5124). Table 1 describes the baseline clinical and biochemical characteristics of the final patient population stratified according to quartile of the serum RDW level at admission (12.7%, 13.4%, and 14.5%). In the final cohort, median age was 58 years (IQR: 47–68 years) and 71.9% were men. Additionally, 68.6% had a NYHA functional class III or IV (*n* = 1975), and 39.7% of patients had coronary heart disease (*n* = 1283) as main diagnosis at discharge. Mean RDW and NT‐proBNP were 13.4% (IQR: 12.7%–14.5%) and 1723.00 pg/ml (IQR: 754.00–4006.25 pg/ml), respectively. The median hemoglobin concentration, creatinine, and high sensitivity CRP (hs‐CRP) were 138.00 g/L (IQR: 124.00–151.00 g/L), 90.30 mmol/L (IQR: 75.85–108.94 mmol/L), and 3.37 mg/L (IQR: 1.54–9.91 mg/L), respectively.

**Table 1:** Characteristics of patients with HF stratified by RDW

Variables	All (*N* = 3231)	RDW < 12.7% (*N* = 705)	12.7% ≤ RDW < 13.4% (*N* = 868)	13.4% ≤ RDW < 14.5% (*N* = 848)	RDW ≥ 14.5% (*N* = 810)	*p *value
Demographics						
Age (years)	58.00 (47.00, 68.00)	56.00 (46.00, 64.00)	59.00 (49.00, 68.00)	59.50 (48.75, 70.00)	58.00 (47.00, 69.00)	<.001
Sex (male), *n* (%)	2323 (71.9)	548 (77.7)	669 (77.1)	600 (70.8)	506 (62.5)	<.001
BMI (kg/m^2^)	24.42 (21.83, 27.24)	25.04 (22.59, 27.43)	25.01 (22.52, 27.75)	23.98 (21.49, 27.05)	23.39 (20.70, 26.49)	<.001
SBP (mm Hg)	119.00 (105.00, 132.00)	120.00 (110.00, 134.00)	120.00 (108.00, 133.00)	118.00 (105.00, 131.00)	114.00 (101.00, 128.00)	<.001
DBP (mm Hg)	70.00 (63.00, 80.00)	71.00 (64.00, 80.00)	71.00 (64.00, 80.00)	70.00 (62.00, 80.00)	70.00 (60.00, 80.00)	.002
Heart rate (beats/min)	78.00 (67.00, 90.00)	75.00 (65.00, 85.00)	76.00 (65.00, 87.25)	78.00 (68.00, 90.00)	83.00 (70.00, 96.00)	<.001
CHD, *n* (%)	1283 (39.7)	339 (48.1)	415 (47.8)	306 (36.1)	223 (27.5)	<.001
NYHA I–II, *n* (%)	902 (31.4)	301 (53.1)	288 (38.9)	199 (25.1)	114 (14.7)	<.001
NYHA III–IV, *n* (%)	1975 (68.6)	266 (46.9)	453 (61.1)	593 (74.9)	663 (85.3)	
HFrEF, *n* (%)	1529 (50.6)	267 (40.8)	393 (48.7)	441 (55.1)	428 (56.2)	<.001
HFmrEF, *n* (%)	506 (16.7)	134 (20.5)	132 (16.4)	134 (16.7)	106 (13.9)	
HFpEF, *n* (%)	989 (32.7)	254 (38.8)	282 (34.9)	226 (28.2)	227 (29.8)	
History of underlying disease						
Hypertension, *n* (%)	1569 (48.6)	362 (51.3)	470 (54.1)	409 (48.2)	328 (40.5)	<.001
Diabetes mellitus, *n* (%)	923 (28.6)	197 (27.9)	281 (32.4)	237 (27.9)	208 (25.7)	.021
Infection, *n* (%)	541 (16.7)	74 (10.4)	123 (14.2)	151 (17.8)	193 (23.8)	<.001
Blood results						
NT‐proBNP (pg/ml)	1723.00 (754.00, 4006.25)	861.00 (436.00, 1839.00)	1357.00 (628.50, 2836.00)	2128.00 (947.00, 4455.00)	3291.00 (1465.00, 6478.50)	<.001
Hs‐CRP (mg/L)	3.37 (1.54, 9.91)	2.25 (0.99, 7.14)	2.96 (1.41, 8.65)	3.55 (1.65, 9.88)	5.40 (2.36, 11.42)	<.001
White cell count (10^9^/L)	7.04 (5.78, 8.58)	6.99 (5.93, 8.38)	7.24 (6.02, 8.79)	6.97 (5.69, 8.41)	6.96 (5.51, 8.54)	.002
Red cell count (10^12^/L)	4.53 (4.10, 5.01)	4.54 (4.20, 4.98)	4.58 (4.19, 5.02)	4.54 (4.07, 5.02)	4.45 (3.86, 5.01)	<.001
Hemoglobin (g/L)	138.00 (124.00, 151.00)	142.00 (131.00, 153.00)	141.00 (128.00, 153.00)	138.00 (124.00, 151.00)	130.00 (112.00, 146.00)	<.001
Hematocrit (%)	41.00 (37.20, 44.90)	41.30 (38.40, 44.60)	41.70 (38.20, 45.10)	41.30 (37.30, 45.10)	39.30 (34.70, 44.50)	<.001
Platelet count (10^9^/L)	195.00 (156.00, 242.50)	206.00 (167.00, 248.00)	194.00 (159.00, 241.00)	190.00 (150.00, 237.00)	192.00 (145.25, 242.00)	<.001
Serum sodium (mmol/L)	138.00 (135.00, 140.07)	138.19 (136.00, 140.00)	138.14 (135.87, 140.52)	138.00 (135.38, 140.41)	136.94 (134.00, 139.70)	<.001
Serum potassium (mmol/L)	3.95 (3.68, 4.27)	3.93 (3.70, 4.18)	3.93 (3.68, 4.22)	3.97 (3.69, 4.29)	4.00 (3.65, 4.31)	.206
Creatine kinase (μmol/L)	90.30 (75.85, 108.94)	85.50 (74.41, 99.50)	89.84 (75.90, 107.50)	93.17 (76.95, 112.85)	93.69 (76.70, 116.15)	<.001
eGFR (ml/min/1.73m^2^)	75.82 (58.92, 93.51)	83.70 (67.88, 97.40)	76.95 (61.86, 93.43)	72.19 (55.29, 89.57)	70.76 (53.35, 89.72)	<.001
Serum uric acid (μmol/L)	418.30 (326.90, 525.18)	374.04 (302.69, 456.76)	408.89 (319.58, 498.52)	430.90 (337.09, 545.48)	473.27 (363.55, 596.16)	<.001
Albumin (g/L)	40.30 (36.90, 43.50)	42.00 (39.10, 44.70)	41.00 (38.10, 44.10)	40.10 (36.80, 43.00)	37.85 (34.00, 41.30)	<.001
Total bilirubin (μmol/L)	19.10 (13.60, 27.10)	16.00 (12.30, 21.21)	17.70 (13.00, 23.40)	20.10 (14.10, 27.71)	25.05 (17.10, 35.02)	<.001
Direct bilirubin (μmol/L)	3.70 (2.50, 5.70)	2.80 (2.10, 3.90)	3.30 (2.30, 4.60)	4.00 (2.60, 6.00)	6.10 (3.60, 9.78)	<.001
Triglyceride (mmol/L)	1.37 (1.03, 1.90)	1.56 (1.17, 2.10)	1.51 (1.12, 2.02)	1.32 (1.00, 1.82)	1.18 (0.90, 1.59)	<.001
Total cholesterol (mmol/L)	4.11 (3.41, 4.91)	4.23 (3.55, 4.96)	4.24 (3.58, 5.06)	4.10 (3.45, 4.93)	3.91 (3.17, 4.68)	<.001
HDL (mmol/L)	0.97 (0.81, 1.19)	1.00 (0.86, 1.21)	0.97 (0.82, 1.18)	0.99 (0.82, 1.24)	0.91 (0.73, 1.11)	<.001
LDL (mmol/L)	2.47 (1.94, 3.10)	2.51 (1.96, 3.12)	2.55 (2.00, 3.19)	2.43 (1.95, 3.12)	2.38 (1.81, 3.00)	.001
Medication						
ACEI or ARB, *n* (%)	1996 (61.8)	493 (69.9)	591 (68.1)	507 (59.8)	405 (50.0)	<.001
Beta blocker, *n* (%)	2785 (86.2)	640 (90.8)	772 (88.9)	714 (84.2)	659 (81.4)	<.001
MRA, *n* (%)	2231 (69.0)	440 (62.4)	560 (64.5)	616 (72.6)	615 (75.9)	<.001
Echocardiography						
LVEF (%)	39.00 (30.00, 55.00)	42.00 (33.00, 56.00)	40.00 (30.00, 55.00)	37.00 (29.20, 51.00)	35.00 (28.00, 53.00)	<.001

*Note*: Data presented as median (Q1, Q3) or *N* (%).

Abbreviations: ACEI, angiotensin converting enzyme inhibitor; ARB, angiotensin II receptor blocker; BMI, body mass index; CHD, coronary heart disease; DBP, diastolic blood pressure; eGFR, estimated glomerular filtration rate; HDL, high density lipoprotein; HF, heart failure; HFmrEF, heart failure with mid‐range ejection fraction; HFpEF, heart failure with preserved ejection fraction; HFrEF, heart failure with reduced ejection fraction; Hs‐CRP, high sensitivity CRP; LDL, low density lipoprotein; LVEF, left ventricular ejection fraction; MRA, mineralocorticoid receptor antagonist; NT‐proBNP, N‐terminal pro‐brain natriuretic peptide; NYHA, New York Heart Association; RDW, red blood cell distribution width; SBP, systolic blood pressure.

Of patients included in this analysis, 50.6% (*n* = 1529) had reduced LVEF (<40%), whereas 506 (16.7%) had moderately reduced LVEF (40%–49%), and 989 (32.7%) had preserved LVEF (≥50%). At discharge, 1996 patients (61.8%) were taking angiotensin‐converting enzyme inhibitors (ACEI) or angiotensin II receptor blockers (ARB), 2785 patients (86.2%) were taking beta‐blockers and 2231 patients (69.0%) were taking mineralocorticoid receptor antagonist (MRA).

With increasing RDW, there was a trend of decreasing hemoglobin and increasing creatinine, hs‐CRP, NT‐proBNP, and proportion of patients with concomitant infections (all *p* < .001). Patients with higher RDW also had lower LVEF (*p* < .001).

### 4.2 Follow‐up

During a 2.9‐year median follow‐up, all‐cause mortality occurred in 1075 (33.27%) patients. The cause of death was classified as cardiovascular in 832 (25.75%), noncardiovascular in 96 (2.97%), and not adjudicated in 253 (7.83%) patients.

### 4.3 RDW for risk prediction: Unadjusted analysis

**Figure 1:**
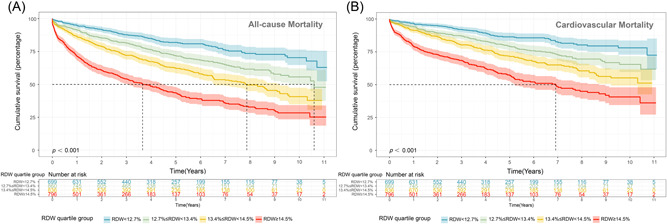
Kaplan–Meier curves for cumulative survival by quartiles of red blood cell distribution width (RDW)

The sample was divided into four groups based on quartiles of RDW. Comparison of Kaplan–Meier survival curves for various quarters over the entire follow‐up period showed good risk stratification (Figure 1) (log‐rank test *p* < .001). All‐cause death occurred in 128 patients (18.1%) in the bottom quarter and 406 (50.1%) in the top quarter (HR: 4.39, 95% confidence interval [CI]: 3.59–5.38; *p* < .001), while cardiovascular death occurred in 87 patients (12.3%) in the bottom quarter and 272 (33.5%) in the top quarter (HR: 4.32, 95% CI: 3.38–5.52; *p* < .001) (Figure 1, Table 2). A similar trend was apparent for 6‐month, 1‐, and 2‐year all‐cause mortality (Supporting Information: Figure S2 and Table S2).

**Table 2:** Cox proportional hazards regression models for all‐cause and cardiovascular mortality

	RDW (continuous variable)			Quarters of RDW				
	HR per 1 SD (95% CI)		*p *value	1	2	3	4	*p* value for trend
				HR (95% CI)	HR (95% CI)	HR (95% CI)	HR (95% CI)	
All‐cause mortality								
Univariate	1.24 (1.21, 1.27)		<.001	1.00	1.64 (1.32–2.05)	2.42 (1.96–2.99)	4.39 (3.59–5.38)	<.001
Model 1	1.25 (1.22, 1.29)		<.001	1.00	1.54 (1.24–1.92)	2.24 (1.82–2.77)	4.32 (3.52, 5.30)	<.001
Model 2	1.20 (1.16, 1.24)		<.001	1.00	1.40 (1.08–1.81)	1.62 (1.26–2.08)	2.96 (2.31–3.78)	<.001
Model 3	1.16 (1.11, 1.21)		<.001	1.00	1.17 (0.88–1.54)	1.24 (0.94–1.62)	2.20 (1.68–2.89)	<.001
Cardiovascular mortality								
Univariate	1.23 (1.19, 1.27)		<.001	1.00	1.67 (1.30–2.20)	2.39 (1.85, 3.08)	4.32 (3.38, 5.52)	<.001
Model 1	1.24 (1.20, 1.28)		<.001	1.00	1.61 (1.24, 2.10)	2.26 (1.75, 2.92)	4.31 (3.37, 5.52)	<.001
Model 2	1.18 (1.13, 1.23)		<.001	1.00	1.42 (1.04, 1.93)	1.54 (1.14, 2.08)	2.73 (2.03, 3.67)	<.001
Model 3	1.15 (1.09, 1.21)		<.001	1.00	1.14 (0.81, 1.61)	1.21 (0.86, 1.68)	2.14 (1.53–2.98)	<.001

*Note*: Model 1 was age and sex adjusted. Model 2 was adjusted for age, sex, BMI, CHD, LVEF, NYHA I–II versus III–IV, eGFR, therapy with ACEI and/or ARB, beta‐blocker, MRA, SBP, heart rate, serum sodium, concomitant infection, combined with diabetes mellitus, combined with hypertension. Model 3 was additionally adjusted for NT‐proBNP. NT‐proBNP were log_2_‐transformed.

Abbreviations: ACEI, angiotensin converting enzyme inhibitor; ARB, angiotensin II receptor blocker; BMI, body mass index; CHD, coronary heart disease; CI, confidence interval; eGFR, estimated glomerular filtration rate; HR, hazard ratio; LVEF, left ventricular ejection fraction; MRA, mineralocorticoid receptor antagonist; NT‐proBNP, N‐terminal pro‐brain natriuretic peptide; NYHA, New York Heart Association; RDW, red blood cell distribution width; SBP, systolic blood pressure.

**Figure 2:**
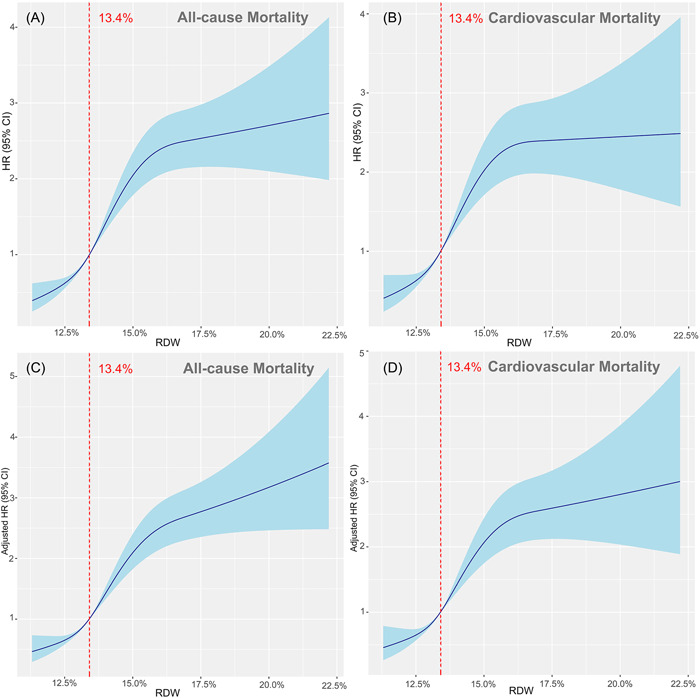
Red blood cell distribution width (RDW) levels and outcome

The unadjusted restricted cubic spline analyses displayed a progressive increase in risk of all‐cause and cardiovascular mortality above the 13.4% RDW threshold (Figure 2A,B), which is the median RDW for the whole population. After adjusting age and sex, the spline curve still showed the same trend, and the inflection points remained at 13.4% for both endpoints (Figure 2C,D).

### 4.4 RDW as independent predictor of outcome

RDW was independently associated with both all‐cause and cardiovascular mortality in models adjusted for age and sex (Model 1), adjusted for additional prognostic indicators of HF (Model 2), as well as in the fully adjusted model that included NT‐proBNP (Model 3) (Table 2). In the fully adjusted model, the risk of all‐cause and cardiovascular mortality in top quarter increased by 120% and 114%, respectively, compared with bottom quarter (both *p*‐trend < .001, Table 2). This association was also apparent in separate analyses of 6‐month mortality, 1‐year mortality, and 2‐year mortality (Supporting Information: Table S2).

**Figure 3:**
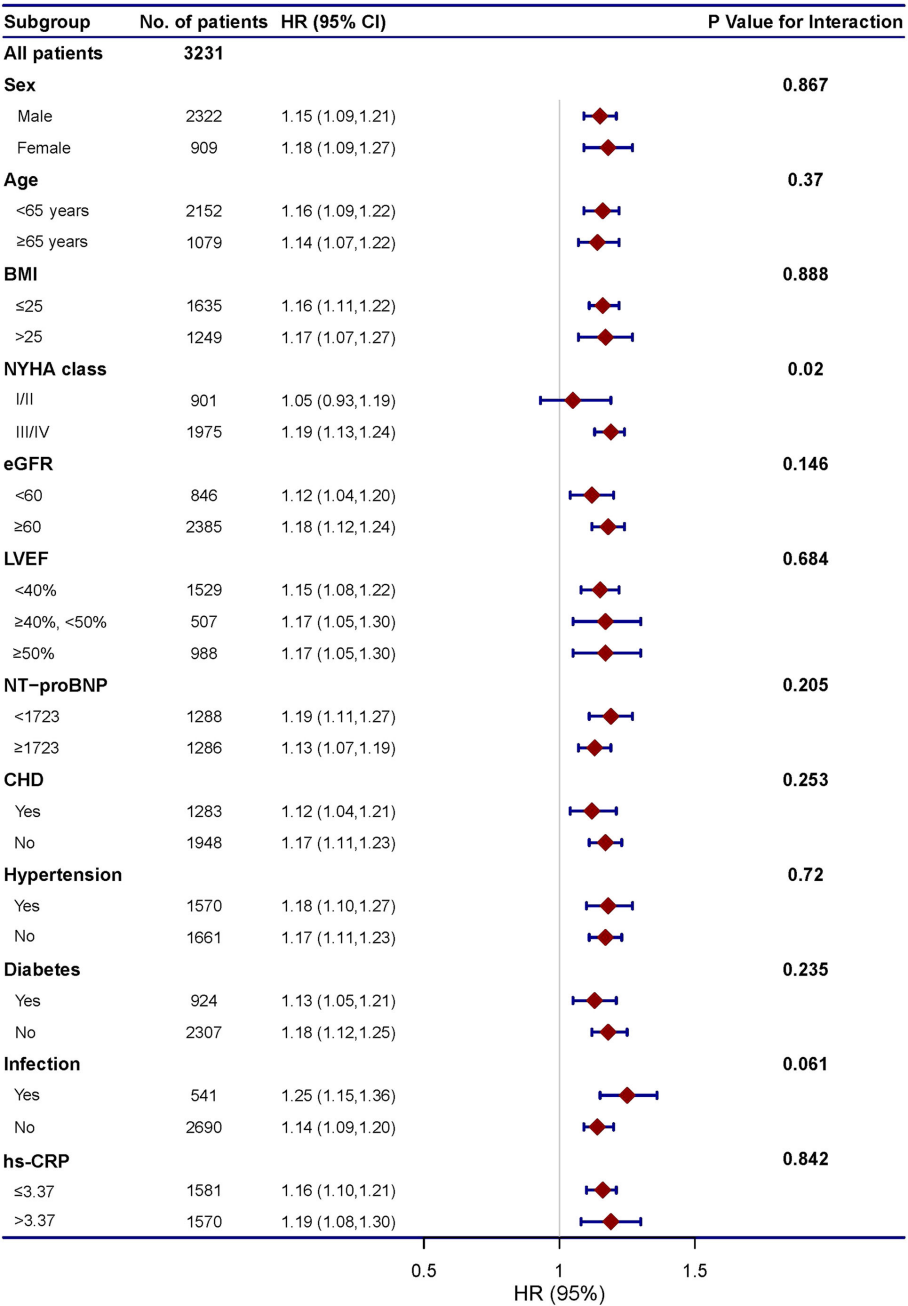
Red blood cell distribution width (RDW) for the prediction of all‐cause mortality: subgroup analysis

Increasing RDW was independently associated with all‐cause mortality across most patient subgroups (Figure 3). Notable exceptions were patients with NYHA I–II. For this subset (*n* = 902), the wide confidence intervals included the HR = 1 line.

### 4.5 NT‐proBNP and RDW concentrations: Clinical and prognostic correlations

According to median NT‐proBNP (1737.00 pg/ml) and RDW (13.4%) concentrations, the included sample was divided into four groups: Low RDW Low NT‐proBNP (RDW < 13.4%, NT‐proBNP < 1737.00 pg/ml; *n* = 852, 33.1%), High RDW Low NT‐proBNP (RDW ≥ 13.4%, NT‐proBNP < 1737.00 pg/ml; *n* = 441, 17.1%), Low RDW High NT‐proBNP (RDW < 13.4%, NT‐proBNP ≥ 1737.00 pg/ml; *n* = 488, 18.9%), and High RDW High NT‐proBNP (RDW ≥ 13.4%, NT‐proBNP ≥ 1737.00 pg/ml; *n* = 793, 30.8%).

**Figure 4:**
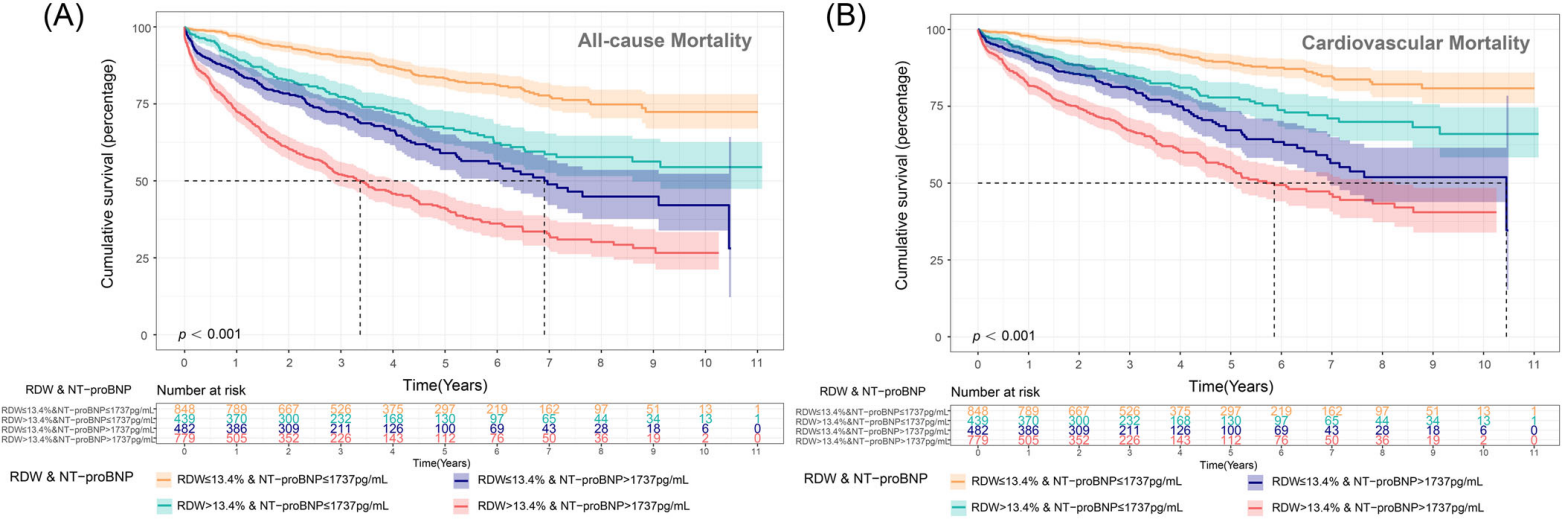
Red blood cell distribution width (RDW) combined with N‐terminal pro‐brain natriuretic peptide (NT‐proBNP) and long‐term prognosis

Of the patients with High RDW High NT‐proBNP, all‐cause mortality occurred in 397 patients (50.1%), as compared with 129 patients (15.1%) with Low RDW Low NT‐proBNP (Figure 4). The Kaplan–Meier survival curve showed a similar trend for 6‐month mortality, 1‐year mortality, and 2‐year mortality (Supporting Information: Figure S3).

For the entire cohort of HF patients, the baseline model (adjusted for age, sex, BMI, CHD, LVEF, NYHA I to II vs. III to IV, eGFR, therapy with ACEI and/or ARB, beta‐blocker, MRA, SBP, heart rate, serum sodium, concomitant infection, diabetes mellitus, hypertension) yielded a C‐index for all‐cause mortality of 0.71, which rose to 0.73 when NT‐proBNP was added. Including RDW led to an increase in C‐statistic when added to the baseline model with, or without, NT‐proBNP (Table 3).

**Table 3:** Model discrimination

Model comparison	C‐index	△C‐index (by adding RDW)
Model 1		
All‐cause mortality	0.71	0.02
Cardiovascular mortality	0.72	0.02
Model 2		
All‐cause mortality	0.73	0.01
Cardiovascular mortality	0.74	0.01

*Note*: Model 1 was adjusted for age, sex, BMI, CHD, LVEF, NYHA I to II versus III to IV, eGFR, therapy with ACEI and/or ARB, beta‐blocker, MRA, SBP, heart rate, serum sodium, concomitant infection, combined with diabetes mellitus, combined with hypertension. Model 2 was additionally adjusted for NT‐proBNP. NT‐proBNP were log_2_‐transformed.

Abbreviations: ACEI, angiotensin converting enzyme inhibitor; ARB, angiotensin II receptor blocker; BMI, body mass index; CHD, coronary heart disease; eGFR, estimated glomerular filtration rate; LVEF, left ventricular ejection fraction; MRA, mineralocorticoid receptor antagonist; NT‐proBNP, N‐terminal pro‐brain natriuretic peptide; NYHA, New York Heart Association; RDW, red blood cell distribution width; SBP, systolic blood pressure.

In terms of predictive accuracy of RDW and NT‐proBNP for all‐cause mortality at any time period, high NT‐proBNP had higher AUCs for the two endpoints, as compared to high RDW (Table 4). The best RDW cutoff for prediction of all‐cause and cardiovascular mortality were 13.45% and 13.65%, respectively. These cutoffs were close to the inflection points of the spline curves (Figure 2), which confirmed the increase in risk above the 13.4% RDW threshold.

**Table 4:** Receiver operator curve analysis: Cutoff values for RDW and NT‐proBNP for mortality

	AUC	Cutoff	Sensitivity	Specificity
All‐cause mortality*				
RDW	0.648	13.45	0.629	0.602
NT‐proBNP	0.677	1737.5	0.686	0.592
Cardiovascular mortality**				
RDW	0.627	13.65	0.572	0.636
NT‐proBNP	0.653	1738.5	0.691	0.565

*Note*: NT‐proBNP was log_2_‐transformed. Area under the curve (AUC) values of NT‐proBNP versus RDW were significantly different for all‐cause mortality.

Abbreviations: NT‐proBNP, N‐terminal pro‐brain natriuretic peptide; RDW, red blood cell distribution width.

* *p* < .05; but insignificant for cardiovascular mortality

** *p* = .1057.

## 5 DISCUSSION

This study confirms RDW levels on admission yield significant and independent prognostic value for predicting all‐cause and cardiovascular mortality in a large cohort of HF patients. Also, concomitant RDW and NT‐proBNP is superior to RDW or NT‐proBNP alone in prognostic assessment. To our knowledge, this is the first time that these associations have been demonstrated for short‐, medium‐, and long‐term outcomes, among different clinical subtypes of HF (HF with reduced ejection fraction [HFrEF], HF with mid‐range ejection fraction [HFmrEF], HF with preserved ejection fraction [HFpEF]), and various patient subgroups (e.g., hypertensive, diabetic). Of special clinical relevance, we suggest cutoff values for RDW and NT‐proBNP to identify those with highest mortality risk.

Despite significant progress in the HF treatment, our ability to monitor and predict the response to therapy remains subpar. Clinical parameters including advanced age, higher NYHA functional class, reduced LVEF, lower body mass index, renal dysfunction, and anemia, confer a poor prognosis.^7^ Recently, biomarkers have changed the way we manage HF patients.^7^, ^12^ BNP and NT‐proBNP are the gold standard biomarkers for confirming the diagnosis and evaluating prognosis in HF, but their clinical application is limited due to challenges in risk stratification.^13^ Specifically, NT‐proBNP levels may be affected by multiple factors, including advanced age, renal insufficiency, and arrhythmias.^14^ Additionally, NT‐proBNP is less useful in HFpEF compared with HFrEF because clinical features of HFpEF, such as atrial fibrillation, obesity, and renal impairment significantly impact NT‐proBNP. Also NT‐proBNP levels may extend into the normal range among HFpEF patients, thereby limiting risk assessment.^15^ This emphasizes the need for alternate HF biomarkers.

RDW is a rapid, inexpensive, and direct hematological parameter, which reflects variability of circulating red blood cell (RBC) size.^16^, ^17^ The prognostic value of RDW has been demonstrated in various subsets of HF patients, in in‐patient and ambulatory settings. For symptomatic HF patients (regardless of EF) enrolled in the North American CHARM program (*n* = 2679), Felker et al.^8^ demonstrated that RDW was a strong independent predictor of morbidity and mortality. These results were replicated in the Duke Databank cohort (*n* = 2140) separately. Among ambulatory CHF patients (*n* = 6159), Cauthen et al.^18^ showed that baseline and 1 year increase in RDW was associated with poor long‐term outcomes. Other smaller studies also reaffirmed the prognostic value of RDW in admitted acute decompensated HF patients.^19^, ^20^

While some of the above studies corrected for natriuretic peptide levels,^18^, ^19^, ^20^ concomitant use of natriuretic peptides and RDW as predictors of HF prognosis was not studied. Furthermore, appropriate cutoff values for RDW specifically for HF patients was not identified, but rather upper limits based on the general population were used. Kawasoe et al.^21^ addressed these shortfalls in a small sample of 116 admitted HF patients. While both RDW and BNP were of independent prognostic significance, considering both RDW and BNP together was superior. Also, the optimal cutoff value for RDW and BNP to predict cardiovascular death were ascertained using receiver operator curve analysis (14.9% and 686 pg/ml, respectively). However, the study was limited by a small sample size, thus limiting the number of variables in the regression analyses.

Our analysis included a large sample, and a central laboratory was used. Herein, we have demonstrated that RDW has a predictive value for cardiac and all‐cause deaths in the short (180 days) medium (1 and 2 years) and long term (10 years). The same conclusion was reached in several population subgroups. RDW was an independent predictor of outcome across all LVEF categories, as well as in patients with or without important comorbidities (diabetes, hypertension, infection, eGFR < 60), and systemic inflammation (i.e., high‐sensitivity C‐reactive protein levels above the median vs. below the median). These findings expand on previous research, in which only patients with reduced EF were included^22^ or different LVEF categories had not been analyzed.^8^ The results also show that the combination of RDW and NT‐proBNP is a more powerful prognostic indicator in HF than either RDW or NT‐proBNP alone. Risk stratification of patients according to both RDW and NT‐proBNP levels may be clinically useful.

The mechanism by which increased RDW predicts prognosis in CHF has not been elucidated. As a measure of variability in circulating erythrocyte size (anisocytosis), RDW is elevated with ineffective RBC production (e.g., iron deficiency, anemia of chronic disease, B12 or folate deficiency, and hemoglobinopathies), increased RBC destruction (e.g., hemolysis), or after blood transfusion.^23^ On one hand, anisocytosis may cause progression of HF and increased mortality. With high anisocytosis, erythrocyte deformability and oxygen‐carrying capacity decrease. This may reduce peripheral and myocardial tissue oxygenation, and contribute to HF.^24^ On the other hand, HF itself may impact RDW. HF involves activation of inflammatory pathways. Inflammatory markers including tumor necrosis factor‐α and interleukin‐6 are increased and may negatively impact prognosis.^25^ Also, inflammation may impair bone marrow function resulting in release of premature erythrocytes into circulation and increased RDW.^24^, ^26^ Impaired iron metabolism, which elevates RDW, is also common in HF and contributes to increased hospitalizations.^27^, ^28^ Vitamin D influences RDW by increasing iron availability and down‐regulating proinflammatory cytokines and hepcidin.^29^ Predictably, vitamin D deficiency is associated with increased risk of HF.^30^, ^31^ Finally, the autonomic nervous system (ANS) plays a regulatory role in bone marrow proliferation. ANS dysregulation, a contributor to HF progression, may also lead to increased RDW.^24^ It is apparent that increased RDW may reflect multiple pathological processes that culminate in HF progression. Summarily, RDW serves as a widely available and cheap way to integrate complex interactions into a single, well validated prognostic biomarker.

Further studies are needed to establish whether RDW or a combination of biomarkers can inform treatment decisions and follow‐up in a cost‐effective manner. At present, even the most established HF biomarkers, the natriuretic peptides, remain controversial for serial follow‐up.^32^, ^33^, ^34^, ^35^ Additionally, the mechanisms behind the prognostic significance of RDW in HF patients merits investigation.

## 6 LIMITATIONS

This study has several limitations. First, it is a retrospective single‐center cohort study, which may affect generalization of results. Second, serial RDW measurements were unavailable, thus, change in RDW could not be assessed. Third, this is an observational study, therefore both causality and directionality are unknown. We do not know if increased RDW causes poor outcomes or whether it is a marker of worse HF. Finally, data on erythropoietin, folate, vitamin B12, and iron levels, which may confound RDW, were unavailable.

## 7 CONCLUSIONS

In conclusion, RDW is an independent predictor of mortality among HF patients in the short‐, medium‐, and long‐term. Combination of RDW and NT‐proBNP improves the prognostic value compared to either alone. This is true across all clinical subtypes of HF (HFrEF, HFmrEF, HFpEF), and among most subgroups of patients with various comorbidities (diabetes, hypertension). Further research into the clinical utility of RDW and the pathophysiology behind its prognostic significance in HF is merited.

## AUTHOR CONTRIBUTIONS

Yuhui Zhang and Jian Zhang designed and supervised the study. Lin Liang, Xuemei Zhao, Lang Zhao, Pengchao Tian, and Boping Huang performed sample and data acquisition. Lin Liang and Liyan Huang performed data analysis and interpretation. Lin Liang wrote the manuscript. Yuhui Zhang and Jian Zhang approved manuscript submission. All authors read and contributed to the manuscript.

## CONFLICT OF INTEREST

The authors declare no conflict of interest.

## Supporting information

Supplementary information.

## Data Availability

The data that support the findings of this study are available on request from the corresponding author. The data are not publicly available due to privacy or ethical restrictions.
