# Supplementary material for: Prognostic value of RDW alone and in combination with NT‐proBNP in patients with heart failure
Source: Clin Cardiol. 2022 May 27;45(7):802–13. doi: 10.1002/clc.23850 (PMC9286336; doi:10.1002/clc.23850)
Supplement: Supplementary file 1 — Supplementary information. [file CLC-45-802-s001.pdf]

SUPPLEMENTAL MATERIAL.

Figure S1 Flowchart illustrating enrollment

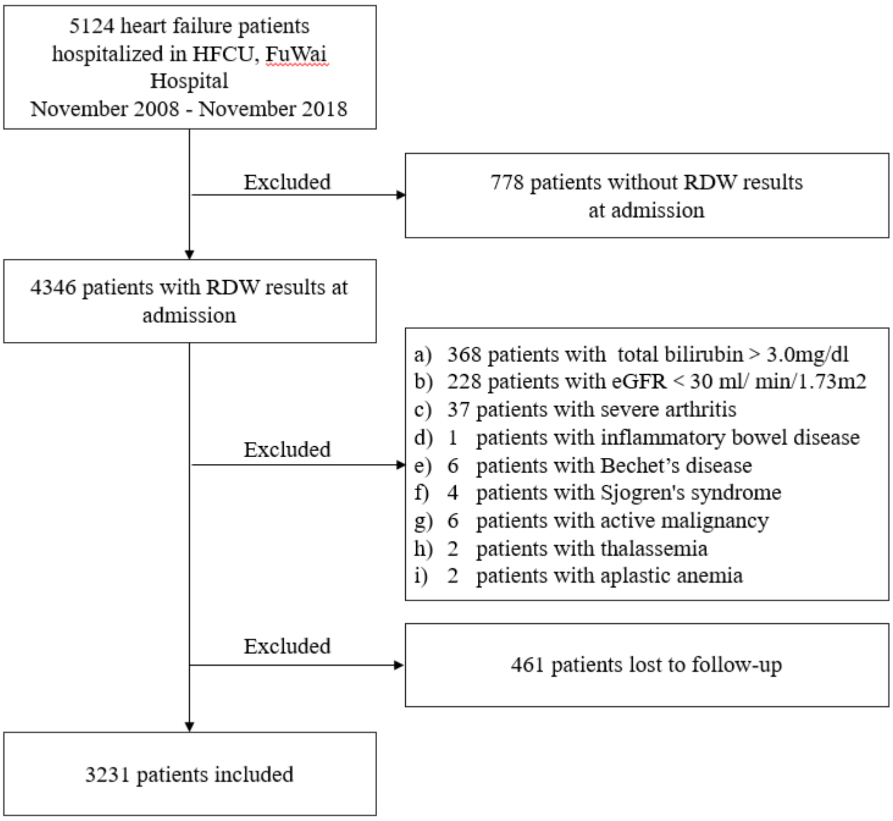

HFCU, heart failure care unit; RDW, red blood cell distribution width; eGFR, estimated glomerular filtration rate

Figure S2 Quartile of RDW and short-to-median term prognosis

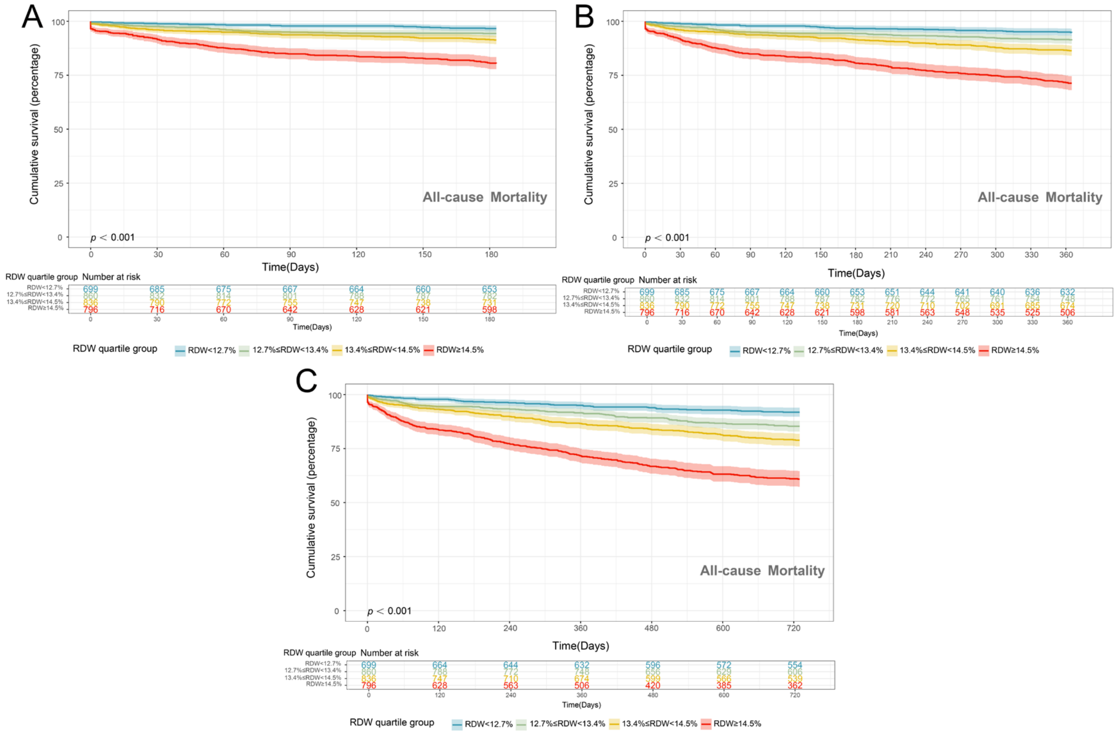

Kaplan-Meier analysis. RDW: red blood cell distribution width.

Figure S3 RDW combined with NT-proBNP and short-to-median term prognosis

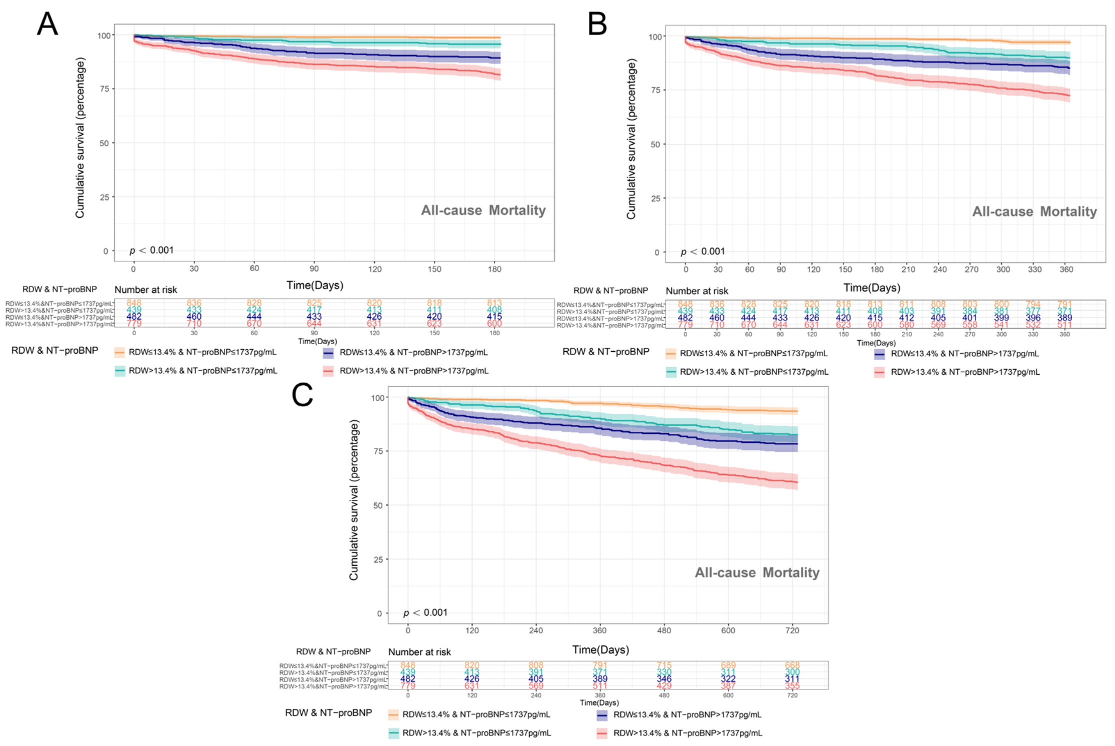

Kaplan-Meier analysis. RDW: red blood cell distribution width; NT-proBNP: N-terminal pro-brain natriuretic peptide.

Table S1 Characteristics of included and excluded patients

Variables	Included Patients N=3231	Excluded Patients N=1893
Demographics		
Age (years)	58.00 [47.00, 68.00]	60.00 [47.00, 71.00]
Sex (male), n (%)	2323 (71.9)	1266 (66.9)
BMI (kg/m^2)	24.42 [21.83, 27.24]	23.80 [20.97, 26.59]
SBP (mmHg)	119.00 [105.00, 132.00]	114.00 [101.00, 130.00]
DBP (mmHg)	70.00 [63.00, 80.00]	70.00 [60.00, 78.00]
Heart rate (beats/min)	78.00 [67.00, 90.00]	78.00 [68.00, 91.00]
CHD, n (%)	1283 (39.7)	790 (41.7)
NYHA I-II, n (%)	902 (31.4)	345 (20.8)
NYHA III-IV, n (%)	1975 (68.6)	1310 (79.2)
HFrEF, n (%)	1529 (50.6)	515 (51.2)
HFmrEF, n (%)	506 (16.7)	148 (14.7)
HFpEF, n (%)	989 (32.7)	343 (34.0)
History of underlying disease		
Hypertension, n (%)	1569 (48.6)	903 (47.7)
Diabetes mellitus, n (%)	923 (28.6)	524 (27.7)
Infection, n (%)	541 (16.7)	379 (20.0)
Medication		
ACEI or ARB, n (%)	1996 (61.8)	1010 (53.4)
Beta blocker, n (%)	2785 (86.2)	1548 (81.8)
MRA, n (%)	2231 (69.0)	1192 (63.0)

Data presented as median [Q1, Q3] or N (%).

BMI, body mass index; SBP, systolic blood pressure; DBP, diastolic blood pressure; CHD, coronary heart disease; NYHA, New York Heart Association; HFrEF, heart failure with reduced ejection fraction; HFmrEF, heart failure with mid-range ejection fraction; HFpEF, heart failure with preserved ejection fraction; ACEI, angiotensin converting enzyme inhibitor; ARB, angiotensin II receptor blocker; MRA, mineralocorticoid receptor antagonist

Table S2 Prognostic value of RDW at various time-points

	RDW (Continuous variable)		Quarters of RDW				
			1	2	3	4	
	HR per 1SD (95% CI)	*P*-value	HR (95% CI)	HR (95% CI)	HR (95% CI)	HR (95% CI)	*p* value for trend
**Six-month mortality**							
Univariate	1.28 (1.22, 1.34)	< 0.001	1.00	1.65 (1.00, 2.73)	2.64 (1.65, 4.23)	6.22 (4.01, 9.66)	<0.001
Model1	1.30 (1.24, 1.36)	< 0.001	1.00	1.64 (0.99, 2.70)	2.65 (1.65, 4.24)	6.44 (4.14, 10.01)	<0.001
Model2	1.19 (1.12, 1.26)	< 0.001	1.00	1.13 (0.62, 2.07)	1.43 (0.82, 2.51)	3.25 (1.92, 5.50)	<0.001
Model3	1.14 (1.05, 1.23)	< 0.001	1.00	0.90 (0.48, 1.68)	0.84 (0.46, 1.53)	1.86 (1.06, 3.26)	<0.001
**One-year mortality**							
Univariate	1.29 (1.24, 1.34)	< 0.001	1.00	1.66 (1.11, 2.48)	2.66 (1.82, 3.87)	6.13 (4.31, 8.73)	<0.001
Model1	1.30 (1.25, 1.35)	< 0.001	1.00	1.63 (1.09, 2.43)	2.61 (1.79, 3.81)	6.20 (4.35, 8.85)	<0.001
Model2	1.21 (1.15, 1.27)	< 0.001	1.00	1.17 (0.74, 1.87)	1.46 (0.94, 2.26)	3.25 (2.15, 4.92)	<0.001
Model3	1.18 (1.11, 1.25)	< 0.001	1.00	0.98 (0.60, 1.62)	1.03 (0.64, 1.65)	2.28 (1.45, 3.58)	<0.001
**Two-year mortality**							
Univariate	1.28 (1.24, 1.32)	< 0.001	1.00	1.82 (1.32, 2.52)	2.74 (2.01, 3.73)	5.88 (4.40, 7.88)	<0.001
Model1	1.30 (1.26, 1.34)	< 0.001	1.00	1.75 (1.27, 2.43)	2.63 (1.93, 3.59)	5.90 (4.40, 7.91)	<0.001
Model2	1.21 (1.17, 1.27)	< 0.001	1.00	1.51 (1.04, 2.20)	1.73 (1.20, 2.49)	3.70 (2.61, 5.24)	<0.001
Model3	1.18 (1.13, 1.24)	< 0.001	1.00	1.24 (0.84, 1.85)	1.29 (0.88, 1.89)	2.68 (1.84, 3.88)	<0.001

Model 1 was age and sex adjusted. Model 2 was adjusted for age, sex, BMI, CHD, LVEF, NYHA I to II vs. III to IV, eGFR, therapy with ACEI and/or ARB, beta-blocker, MRA, SBP, heart rate, serum sodium, concomitant infection, combined with diabetes mellitus, combined with

hypertension. Model 3 was additionally adjusted for NT-proBNP. NT-proBNP were log2-transformed. HR = hazard ratio; CI = confidence interval; other abbreviations as in Table 1.
